# Quantum Dot-Based Molecular Beacon to Monitor Intracellular MicroRNAs

**DOI:** 10.3390/s150612872

**Published:** 2015-06-02

**Authors:** Jonghwan Lee, Sung Ung Moon, Yong Seung Lee, Bahy A. Ali, Abdulaziz A. Al-Khedhairy, Daoud Ali, Javed Ahmed, Abdullah M. Al Salem, Soonhag Kim

**Affiliations:** 1 Institute for Bio-Medical Convergence, College of Medicine, Kwandong Catholic University, Gangneung-si, Gangwon-do 270-701, Korea; E-Mails: jonghwanlee104@gmail.com (J.L.); mboy2@korea.com (S.U.M.); inyasio0731@naver.com (Y.S.L.); 2 Catholic Kwandong University International St. Mary’s Hospital, Incheon Metropolitan City 404-834, Korea; 3 Aljeraisy DNA Research Chair, Department of Zoology, College of Science, King Saud University, Riyadh 11451, Saudi Arabia; E-Mails: bahali@ksu.edu.sa (B.A.A.); javedbiochem@gmail.com (J.A.); alsalem1985@hotmail.com (A.M.A.); 4 Department of Nucleic Acids Research, Genetic Engineering and Biotechnology Research Institute, City for Scientific Research and Technological Applications, Alexandria 21934, Egypt; 5 Department of Zoology, College of Science, King Saud University, Riyadh 11451, Saudi Arabia; E-Mails: kedhairy@ksu.edu.sa (A.A.A-K.); daoudali_p@yahoo.com (D.A.)

**Keywords:** quantum dot, microRNA, molecular beacon, neurogenesis

## Abstract

Fluorescence monitoring of endogenous microRNA (miRNA or miR) activity related to neuronal development using nano-sized materials provides crucial information on miRNA expression patterns in a noninvasive manner. In this study, we report a new method to monitor intracellular miRNA124a using quantum dot-based molecular beacon (R9-QD-miR124a beacon). The R9-QD-miR124a beacon was constructed using QDs and two probes, miR124a-targeting oligomer and arginine rich cell-penetrating peptide (R9 peptide). The miR124a-targeting oligomer contains a miR124a binging sequence and a black hole quencher 1 (BHQ1). In the absence of target miR124a, the R9-QD-miR124a beacon forms a partial duplex beacon and remained in quenched state because the BHQ1 quenches the fluorescence signal of the R9-QD-miR124a beacon. The binding of miR124a to the miR124a binding sequence of the miR124a-targeting oligomer triggered the separation of the BHQ1 quencher and subsequent signal-on of a red fluorescence signal. Moreover, enhanced cellular uptake was achieved by conjugation with the R9 peptide, which resulted in increased fluorescent signal of the R9-QD-miR124a beacons in P19 cells during neurogenesis due to the endogenous expression of miR124a.

## 1. Introduction

Endogenous microRNAs (miRNA or miR) are single-stranded, non-coding RNA molecules responsible for governing gene expression. Investigation of the expression level of these miRNAs has occupied a vital position in the understanding of a wide range of biological processes such as cellular proliferation, differentiation, death, and disease. Real-time PCR, northern blot analysis, and fluorescence *in situ* hybridization (FISH) analysis have been widely used at the cellular level to detect the cellular distribution and expression level of intracellular miRNAs [1,2,3]. Unfortunately, these methods require cell destruction and do not provide time course information of miRNA expression in living organisms [4,5]. Therefore, the development of a detection method that tracks intracellular miRNAs in living cells is required.

Recently, a 3′-untranslated region (UTR)-based reporter gene imaging system has been successfully developed to monitor the expression patterns of mature miRNAs [6,7,8,9]. However, due to the miRNA function that binds to sequences that are partially or completely complementary to mature miRNAs and then degrades mRNA or inhibits translation, the reporter gene-based miRNA detection system is accompanied by signal-off results. In a signal-off system, it is difficult to distinguish whether the signal-off data results from substantial miRNA expression or only from cell death *in vivo*. Therefore, a signal-on imaging system is technically required to overcome the shortcoming of the conventional signal-off reporter imaging system and monitor intracellular miRNAs. 

Molecular beacons offer a powerful approach to real-time visualization of specific endogenous mRNAs and simultaneous monitoring of gene expression in cancer cells [10,11,12,13]. Therefore, a molecular beacon strategy might be suitable for the detection of expression levels of endogenous small molecules in living subjects. The quencher-based molecular beacon system, with an on/off fluorescence signal, is easily controlled by sequence-sequence matching and is well-suited for detection of expression profiles of endogenous miRNAs. Molecular imaging approaches based on nanotechnology have played an important role in the non-invasive tracking of cancer or drugs using nano-sized materials like semiconductor quantum dots (QDs) as cancer targeted carriers. QDs have been broadly applied to biological imaging as fluorescence probes. They have several advantages, including a high quantum yield, size-dependent tunable emissions, and photo-stabilizing effects against photo-bleaching [14,15]. The broad application of fluorescence QDs to biological techniques has been achieved, including the detection of target proteins using a molecular beacon system coupled with QDs [16]. 

**Figure 1:**
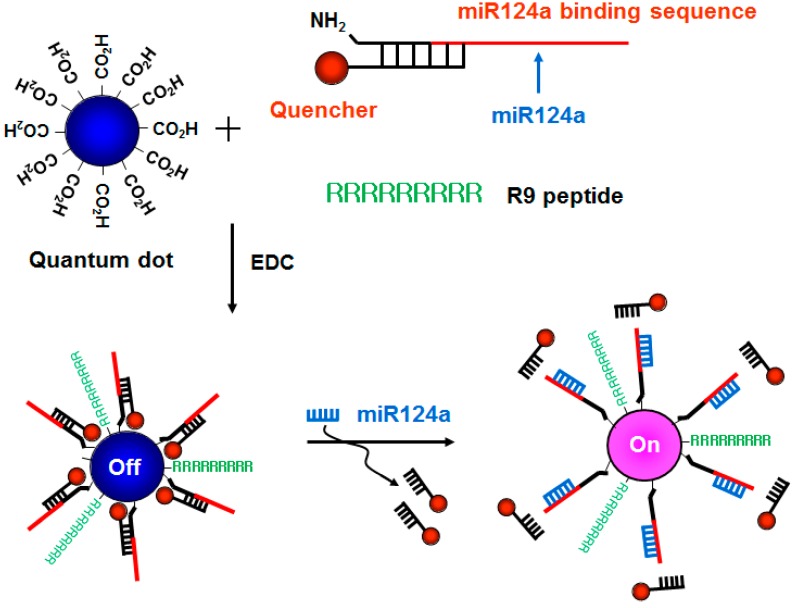
A schematic diagram of the R9-QD-miR124a beacons to image miR124a. The amine-terminated oligonucleotide containing the miR124a recognition sequence was annealed at the region with partial complementary sequences attached to the quencher molecules, which was designated as the miR124a-targeting oligomer. The carboxyl-terminated QD655 was conjugated with the miR124a-targeting oligomer and the R9 peptide. When the mature miR124a was hybridized with the miR124a binding region in the R9-QD-miR124a beacons, the quencher molecules were separated, resulting in an increase in the fluorescence signal.

Here, we developed a new method for the fluorescence imaging of endogenous miRNAs using a signal-tunable molecular beacon technique based on QDs in living cells. To improve cellular uptake, a 9-mer arginine-rich peptide was attached to QDs. We targeted miRNA124a, which is known to be specifically and highly expressed in neurons and to have a well-established expression profile during neuronal development [17]. In the absence of miR124a, the R9-QD-miR124a beacon forms a partial duplex beacon that quenches the system and leaves QDs located in adjacent regions to each other (Figure 1). No fluorescence signal is observed in this situation. In contrast, the presence of miRNA124a separates the quencher molecules from the R9-QD-miR124a beacon, resulting in signal-on of a red fluorescence signal. The recovery of fluorescence signal is closely dependent on the target miR124a concentration because the extent of separation of the quencher molecule on the R9-QD-miR124a beacon is affected by the number of target miR124a.

This method could provide valuable information by monitoring endogenous miR124a expression during neurogenesis. Furthermore, we expect that the QD-based molecular beacon strategy could be applied to high-throughput imaging systems that evaluate the molecular network of cellular developments and diseases by simultaneously monitoring a variety of endogenous miRNAs using multiple, differently sized QD-based molecular beacons systems in living organisms. 

## 2. Experimental Section

### 2.1. Preparation and Characterization of the miR124a-Targeting Oligomer and the 9-Mer Arginine-Rich Peptide-Conjugated Quantum Dots (R9-QD-miR124a Beacons)

The miR124a-targeting oligomer, a partially double-stranded oligomer composed of a miR124a binding sequence and a black hole quencher 1 (BHQ1), was synthesized by Bionics (Bionics, Inc. Seoul, Korea). The oligomer sequences were as follows: the 3′-adaptor oligomer was 5′-NH_2_-TTCGCTGTTGGCATTCACCGCGTGCCTTAA-3′, and the 5′-adaptor oligomer was 5′-TGCCAACAGCG-BHQ1-3′. The underlined regions in the 3′-adaptor represent the miR124a binding site. The 10 pM of carboxyl-terminated quantum dots (QD655 ITK Carboxyl Quantum Dots; Invitrogen, Grand Island, NY, USA) were coupled with the 300 pM of miR124a-targeting oligomer and the 2 nM of 9-mer arginine peptide (R9 peptide) using 0.2 mM of *N*-(3-dimethylaminopropyl)-*N'*-ethyl-carbodiimide hydrochloride (EDC, Sigma-Aldrich, St Louis, MO, USA) in 25 mM 4-morpholineethanesulfonic acid monohydrate (MES) buffer (pH 6.5) for 1 h at room temperature. Unconjugated reactants were removed by centrifugation at 22,250 g for 30 min. 

Transmission electron microscopy (TEM; JEM 1010, JEOL, Tokyo, Japan) image analysis was conducted to evaluate the size and water dispersion of the prepared R9-QD-miR124a beacons. Electrophoretic shift assay was performed to confirm the conjugation reaction. Unconjugated QDs and the R9-QD-miR124a beacons were loaded onto a 2% agarose gel to analyze their mobility shift. Dynamic light scattering was conducted to compare the size distribution of the QDs and the R9-QD-mir124a beacons using Zetasizer Nano ZS (Malvern Instruments, Worcestershire, UK). The coupling efficiency between QDs and the miR124a-targeting oligomer or the R9 peptide was calculated by measuring the oligomer or peptide concentrations before and after coupling reaction from the supernatant after centrifugation of the reaction mixtures using NanoDrop ND-1000 Spectrometer (Thermo Fisher Scientific, Wilmington, DE, USA).

### 2.2. Cell Culture

C6 rat glioma cells were maintained in DMEM (Gibco, Grand Island, NY, USA) supplemented with 10% fetal bovine serum (FBS; Invitrogen), 10 U/mL penicillin (Invitrogen), and 10 μg/mL streptomycin (Invitrogen) in a 5% CO_2_-humidified chamber at 37 °C. P19 cells (mouse embryonic carcinoma cell line) were cultured in DMEM supplemented with 10% FBS, 10 U/mL penicillin, 10 μg/mL streptomycin, 1% non-essential amino acid solution (NEAA; Sigma-Aldrich), and β-mercapto-ethanol (Sigma-Aldrich). To induce neuronal differentiation of the P19 cells, the cells were cultured in DMEM/F12 (1:1) medium (Gibco) supplemented with N2 supplement (Gibco) and 0.5 mM all-trans retinoic acid (RA; Sigma-Aldrich). Two days later, medium was replaced by fresh medium without RA.

### 2.3. Internalization of the R9-QD-miR124a Beacons into Cells and Imaging of miR124a

The cells (1 × 10^5^) were seeded into 24-well plates and maintained for 18 h. Then, incubated cells were treated with the R9-QD-miR124a beacons and incubated at 37 °C for 1 h. To monitor exogenous miR124a expression in C6 cells, the synthetic miRNA124a precursors were transfected into the cells using Lipofectamine (Invitrogen). Before treatment of miRNA124a precursors, several washing steps with PBS were performed to minimize binding competition between added miRNA124a precursors and the retained R9-QD-miRNA124a beacons in the culture medium. To detach the cells from culture dish, lysis solution containing Triton X-100 was used for protein normalization. The harvested cells were transferred into 96-well black plates for the acquisition of fluorescence signals by a fluorescence imager (Varioskan Flash, Thermo Fisher Scientific, Vantaa, Finland).

### 2.4. Cytotoxicity of the R9-QD-miRNA124a Beacons

C6 and P19 cells were cultured in 96-well plates for 24 h at the seeding density of 5 × 10^3^/well. Different concentrations (0, 5, 10, 20, and 50 pM) of the R9-QD-miRNA124a beacons and the QD-miR124a beacons were added and cells were further cultured. After 24 h, cells were treated with 3-(4,5-dimethythiazolyl)-2,5-diphenyletrazolium bromide (MTT) solution and cultured for 4 h. Followed by dissolving formazan crystals using dimethyl sulfoxide (DMSO), absorbance was measured at 570 nm using microplate reader (Microplate Reader 680, BioRad, Hercules, CA, USA).

### 2.5. Quantitation of Endogenous miR124a Expression in P19 Cells

The endogenous miR124a expressed during neurogenesis of the P19 cells was analyzed by quantitative reverse polymerase chain reaction (qRT-PCR). Total RNA was purified using TRIzol^®^ Reagent (Invitrogen). The miR-Q assay was conducted using 200 ng RNA for miRNA qRT-PCR. The 5S rRNA was used as a reference to analyze the relative expression level of miR124a. Student’s t-test was used for statistical analysis.

### 2.6. The Confocal Laser Scanning Microscope Analysis

The cells (1 × 10^5^), grown in 24-well plates containing gelatin-coated glass coverslips, were fixed with 3.7% paraformaldehyde (Sigma-Aldrich) and mounted onto the glass slide containing VECTASHIELD^®^ Mounting Medium with DAPI (Vector Laboratories, Inc., Burlingame, CA, USA). For imaging of neurogenesis in P19 cells, paraformaldehyde-treated P19 cells were further treated with 0.25% Triton X-100 (in PBS). After blocking with 5% (w/v) normal goat serum and 0.2 (v/v) Tween-20, cells were incubated with anti-Oct3/4 (Chemicon, Millipore, Watford, UK) or anti-NeuroD (Chemicon). Followed by several washings with PBS, Alexa Fluor^®^ 594 conjugated secondary antibody (Abcam, Cambridge, MA, USA) was added. The fluorescence images were aquired by confocal laser scanning microscopy (LSM 510; Carl Zeiss, Oberkochen, Germany).

### 2.7. Fluorescence Intensity

P19 cells were grown in 24-well plates at a seeding concentration of 5 × 10^5^ cells per well. The R9-QD-miR124a beacons were added and further cultured for 1 day. And then, neuronal differentiation of P19 cells was induced. During the culture period, cells were washed and lysed with radio-immunoprecipitation assay (RIPA) buffer (Thermo Fisher Scientific Inc., Waltham, MA, USA). Fluorescence intensity was analyzed using a BioTek Fluorescent Microplate Fluorometer (Synergy Mx, BioTeck Ltd., Winooski, VT, USA).

## 3. Results

### 3.1. Characterization of the R9-QD-miR124a Beacons

**Figure 2:**
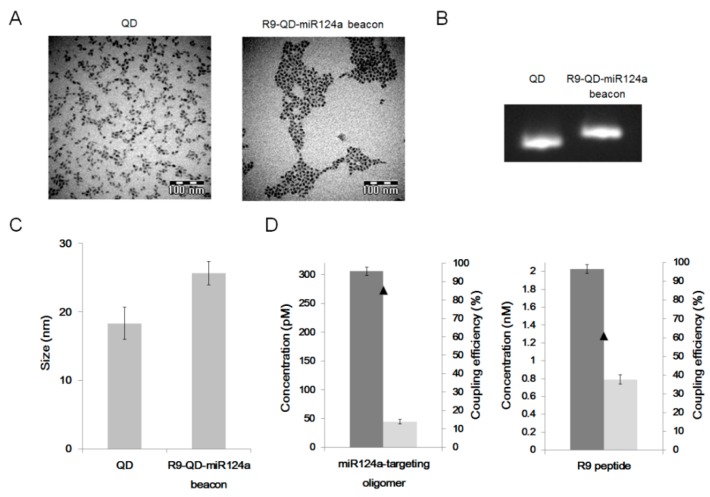
Characterization of the R9-QD-miR124a beacons. (**A**) Transmission electron microscopy (TEM) images; (**B**) electrophoretic shift assay; and (**C**) dynamic light scattering (DLS) analysis of the unconjugated QDs and the R9-QD-miR124a beacons; (**D**) Coupling efficiencies of the miR124a-targeting oligomer and the R9 peptide to QDs.

The miR124a-targeting oligomer and the R9 peptide were covalently conjugated with the QD655 through EDC coupling. A brief sonication was applied to the mixture to disperse the aggregated R9-QD-miR124a beacons. TEM imaging confirmed that the prepared R9-QD-miR124a beacons were fully dispersed in Tris buffer (pH 7.4) (Figure 2A). Electrophoretic shift assay showed the mobility shift between the unconjugated QDs and the prepared R9-QD-miR124a beacons (Figure 2B). DLS analysis further demonstrated that the average hydrodynamic sizes of the QDs and the R9-QD-miR124a beacons were 19 and 26 nm, respectively (Figure 2C). The size distinction between the QDs and the R9-QD-miR124a beacons implies a successful conjugation. The coupling efficiency of the miR124a-targeting oligomer and the R9 peptide to QDs was 85.3% and 60.9%, respectively (Figure 2D).

### 3.2. Specificity of the R9-QD-miR124a Beacons for miR124a

**Figure 3:**
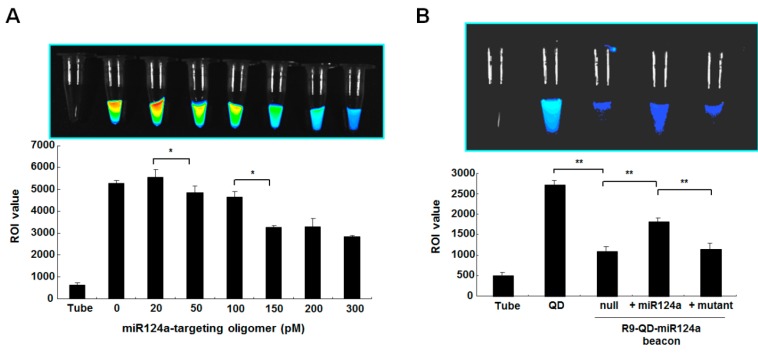
(**A**) Quenching efficiency of the R9-QD-miR124a beacons in the tube. A fixed concentration of the QDs (10 pM) were conjugated with various concentrations (0–300 pM) of the miR124a-targeting oligomer to determine the optimal concentration of miR124a needed to achieve the best quenching effect. The fluorescence activity of the R9-QD-miR124a beacons gradually decreased as the concentration of miR124a-targeting oligomer increased. ROI analysis from the fluorescence tube image showed that the fluorescence signal decreased in a dose-dependent manner (* *p* < 0.05); (**B**) The fluorescence recovery effect of the R9-QD-miR124a beacons after treatment of an exogenous miR124a in the tube. The R9-QD-miR124a beacons were treated with an exogenous (300 pM) or a mutant (300 pM) miR124a for 1 h at 37 °C. The quenched fluorescence intensity was activated in the conjugate group in the presence of the miR124a; however, no fluorescence recovery was observed in the mutant oligonucleotide group (** *p* < 0.005).

The optimal quenching efficiency was examined by incubation of a fixed concentration of the QDs (10 pM) with increasing concentrations (0–300 pM) of miR124a-targeting oligomers. At miR124a-targeting oligomer concentrations above 150 pM, the fluorescence intensity of the R9-QD-miR124a beacons in the tube was gradually quenched (58.4%) (Figure 3A). This finding suggests that the conjugation reaction for preparation of the R9-QD-miR124a beacons should be conducted with more than 150 pM of the miR124a-targeting oligomers. 

The fluorescence recovery of the quenched R9-QD-miR124a beacons was analyzed using synthetic exogenous miR124a. Incubation of the prepared R9-QD-miR124a beacons with exogenous miR124a (300 pM) for 1 h induced the displacement of quencher oligo and a concurrent increase in fluorescence from the R9-QD-miR124a beacons (Figure 3B). However, incubation of the R9-QD-miR124a beacons with either a null or mutant (300 pM of mutated miR124a) sample did not show any significant change in fluorescence intensities. The R9-QD-miR124a beacons incubated with negative controls still maintained the stabilized partial duplex structure between the miR124a binding region and the quencher oligo, resulting in quenching of the fluorescence. These results proved the high specificity of the R9-QD-miR124a beacons for detecting miR124a.

**Figure 4:**
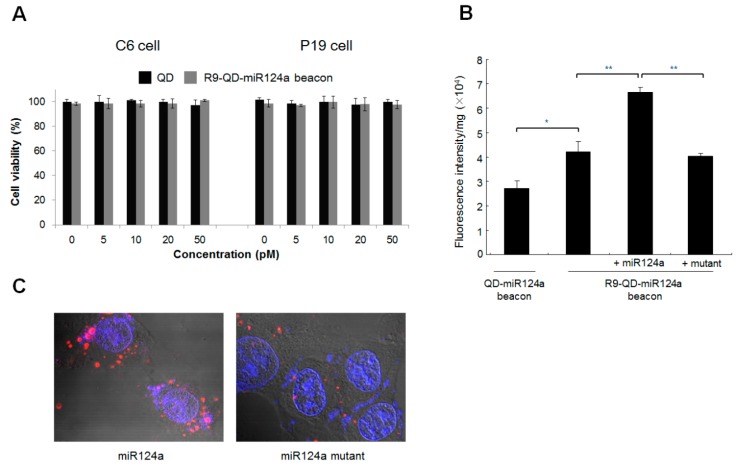
The activation of fluorescence intensity of the R9-QD-miR124a beacon in C6 cells. (**A**) *In vitro* cytotoxicity of QDs and the R9-QD-miR124a beacons in cells; (**B**) R9 peptide conjugation improved internalization efficiency and miR124a specificity of the R9-QD-miR124a beacons in C6 cells. The fluorescence signal from the quenched R9-QD-miR124a beacons was activated in the exogenous miR124a-treated group (300 pM), unlike in the mutant-treated group (300 pM); (**C**) The visualization of fluorescence recovery of the R9-QD-miR124a beacons in C6 cells. The R9-QD-miR124a beacons were incubated with C6 cells for 1 h 30 min, and exogenous miR124a or the miR124a mutant was added to the pre-treated C6 cells. Confocal microscopy imaging showed that the fluorescence signal of the R9-QD-miR124a beacons was significantly activated in C6 cells after treatment with exogenous miR124a. In contrast, induction of the miR124a mutant showed a weak fluorescence signal of the R9-QD-miR124a beacon in C6 cells, suggesting maintenance of the quenched fluorescence signal.

### 3.3. Imaging of Exogenous miR124a in C6 Cells

To image exogenous miR124a expression in cells, C6 cells were treated with the R9-QD-miR124a beacons and transfected with exogenous miR124a using Lipofectamine. C6 cells were chosen because they do not express endogenous miR124a [18]. To compare internalization efficiency, the R9 peptide-nonconjugated QD-miR124a beacons were also used. Unmodified QDs and R9-QD-miR124a beacons did not affect C6 and P19 cells viability (Figure 4A). After 3 h of incubation, the R9-QD-miR124a beacons showed one and a half times increased fluorescence intensity (Figure 4B). This finding may indicate that the different surface coverage of the miR124a-targeting oligomers conjugated to the QD-miR124a beacons and the R9-QD-miR124a beacons. Unlike mono-miR124a-targeting oligomer-functionalized QD-miR124a beacons, the dual-functionalized R9-QD-miR124a beacons have relatively low miR124a-targeting oligomer density on QD surfaces. It can yield a lower degree of fluorescence quenching, finally resulting in increased fluorescence intensity. This finding also demonstrates that the enhanced cellular uptake of the R9-QD-miR124a beacons was achieved by conjugation with R9 peptide. Such arginine-rich cell-penetrating peptides have been extensively studied as carriers for enhanced cellular uptake [19,20,21]. The enhanced cellular uptake further enabled the specific imaging of an exogenous miR124a (300 pM) in C6 cells. However, incubation of the R9-QD-miR124a beacons with null or mutant samples in C6 cells maintained the quenched state. Confocal microscope images further confirmed that the introduction of exogenous miR124a into the C6 cells treated with the R9-QD-miR124a beacons activated the fluorescence signal more than when miR124a mutant-treated C6 cells were treated with the R9-QD-miR124a beacons (Figure 4C). 

### 3.4. Imaging of Endogenous miR124a during Neuronal Differentiation of P19 Cells

**Figure 5:**
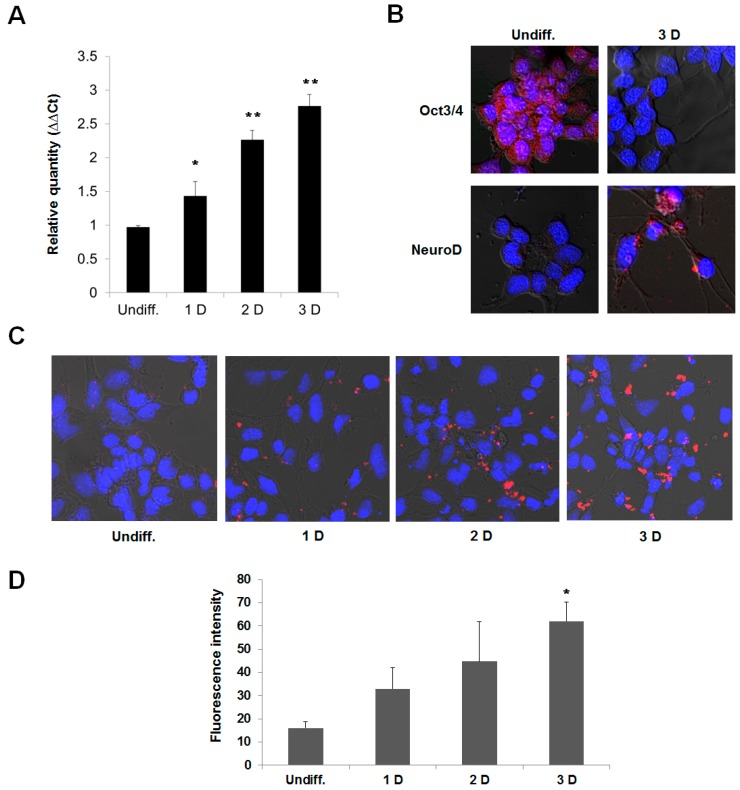
The activation of fluorescence intensity of the R9-QD-miR124a beacons in differentiated P19 cells. The P19 cells were induced to neuronal differentiation for 3 days. (**A**) qRT-PCR analysis of the expression of miR124a during neuronal differentiation in the P19 cells; (**B**) Immunocytostaining of the P19 cells by Oct3/4 (stem cell marker) and NeuroD (neuronal marker) antibodies. Red fluorescence indicates Oct3/4 or NeuroD expression, and blue fluorescence indicates DAPI, which stains the nucleus; (**C**) Undifferentiated and differentiated P19 cells were incubated with the R9-QD-miR124a beacons. Confocal microscopy imaging showed that the fluorescence signal of the R9-QD-miR124a beacons in differentiated P19 cells was significantly activated by endogenous miR124a. Images were merged with DAPI; (**D**) Fluorescence intensity of the R9-QD-miR124a beacons in P19 cells during neuronal differentiation. Data are displayed as means ± standard error of triplicate samples (* *p* < 0.05, ** *p* < 0.005).

To test whether the R9-QD-miR124a beacons are available to image endogenous miR-124a expression during neurogenesis, the R9-QD-miR124a beacons were transferred into undifferentiated and differentiated P19 cells. The R9-QD-miR124a beacons did not affect the viability of P19 cells (Figure 4A). RT-PCR analysis showed increased expression of endogenous miR124a during neuronal differentiation of the P19 cells (Figure 5A). Neurogenesis was further confirmed by immunocytochemistry showing decreased expression of Oct3/4 and increased expression of NeuroD during the 3 days of neuronal differentiation (Figure 5B). Unlike undifferentiated P19 cells, P19 cells after the induction of neuronal differentiation by RA demonstrated activated fluorescence signal of the R9-QD-miR124a beacons (Figure 5C). Similarly to the increased expression or miR124a, fluorescence intensity of the R9-QD-miR124a beacon in signal-on mode also increased during the neuronal differentiation (Figure 5D). These results suggest that the activated fluorescence signal of the R9-QD-miR124a beacons in differentiated P19 cells was caused by endogenous miR124a expression.

## 4. Discussion

The development of nanotechnologies has led to the growth of molecular imaging and diagnostics. The non-invasive detection and quantitation of miRNAs in living organisms has been frequently requested as a bioimaging tool. Semiconductor QDs have been employed for a variety of biochemical applications to sense biomolecules and for delivery of extracellular materials because of the ease of synthesis and their unique optical properties. Moreover their nano-sized structure offers an increased surface area for immobilization of targeting or imaging probes such as nucleotides, aptamers, receptors, and proteins. Despite these benefits, the application range of these probes is sometimes limited because of the difficulty of distinguishing between targets and other molecules. To minimize such nonspecific targeting and improve target specificity, we employed a quencher-dependent on/off imaging platform using QD-based molecular beacon. The activation of fluorescence signal from the quenched R9-QD-miR124a beacons was induced by target miR124a binding and subsequent quencher detachment. This method would be valuable for imaging several miRNAs and diagnosing their associated cellular diseases.

## 5. Conclusions/Outlook

This study describes a platform of QD-based imaging of miRNA biogenesis during differentiation. For target miR124a imaging, the miR124a-targeting oligomers were conjugated to QDs. Functionalization of QDs was confirmed by fluorescence detection using exogenous and mutant miR124a. Enhanced cellular uptake was achieved by functionalization of the R9 peptide onto the QDs. Imaging of endogenous miR124a expression was performed by treating undifferentiated and differentiated P19 cells with the R9-QD-miR124a beacons. This QD-based signal-on method can successfully be used for *in vitro* and *in vivo* monitoring and imaging of intracellular miRNAs.
